# Pharmacotherapeutic Patterns and Patients’ Access to Pharmacotherapy for Some Rare Diseases in Bulgaria – A Pilot Comparative Study

**DOI:** 10.3389/fphar.2021.695181

**Published:** 2021-07-19

**Authors:** Maria Kamusheva, Maria Dimitrova, Konstantin Tachkov, Guenka Petrova, Zornitsa Mitkova

**Affiliations:** Department of Organization and Economics of Pharmacy, Faculty of Pharmacy, Medical University-Sofia, Sofia, Bulgaria

**Keywords:** rare diseases, guidelines, Bulgaria, orphan drugs, market entrance

## Abstract

Provision of the latest innovative and advanced therapies for rare diseases (RDs) patients, following the international therapeutic recommendations, is crucial and necessary for both practitioners and patients. The goal is to assess the access of Bulgarian patients with the most cost-consuming RDs to medicines and to compare the pharmacotherapeutic patterns in Bulgaria and the relevant European professional associations. Pharmaco-therapeutic guidelines for treating the most cost-consuming RDs in Bulgaria were analyzed to assess their compliance with the European ones. Market entrance was evaluated through analysis of the availability of medicines in the Positive Drug List (PDL) and their date of inclusion since marketing authorization. Guidelines’ compliance index was calculated and patient access was analyzed through evaluation of the National Health Insurance Fund (NHIF) standards, which provide additional criteria for treatment initiation. The analyzed guidelines follow the adopted recommendations by the relevant European professional associations. NHIF have exclusion and inclusion criteria for initiating treatment with medicines for rare diseases and for continuation. The average time-lag between centralized procedure approval and inclusion in the Bulgarian PDL for orphan medicinal products (MPs) is 6.75 years (SD = 4.96) with the longest time observed for eptacog alfa (20 years) and the shortest for rurioctocog alfa pegol, octocog alfa and simoctocog alfa (1 year). Bulgarian patients with cystic fibrosis with pulmonary manifestation had a wait time of only 1.6 years to get access to innovative, centrally authorized medicines, whereas the period for access to acromegaly treatment was 8.2 years. The main factors influencing market entrance and patient access are the time to inclusion in the PDL and the NHIF criteria.

## Introduction

Pharmacotherapy and clinical practice guidelines are developed and incorporated by Professional societies in order to meet the requirements for precise and quality medical care (Field and Lohr, 1990; Heins et al., 2017). Following the latest studies, these guidelines provide evidence-based procedures regarding the diagnosis, care and available treatment and giving the possibility to practitioners to choose the appropriate and most suitable therapy for their patients (Heins et al., 2017). In this way, therapeutic outcomes could be improved by encouraging prescription of proven effective treatments and discouraging those without proven effectiveness and safety (Heins et al., 2017). Each recommendation included in the guidelines could be classified as strong or weak depending on its importance and the amount of scientific evidence behind it. The quality of the evidence used as a basis for the recommendations could be graded as high, moderate or low based on the quality of the performed studies (Australian and International Guidelines on Diabetic Foot Disease, 2016).

It should be noted that, in light of the increasing scientific progress in the medical and pharmaceutical area, and with the amount of new evidence generated, there is a risk of recommendations becoming out-of-date (Heins et al., 2017). To prevent this, expert working groups within the medical professionals’ associations regularly update these guidelines, which are then internationally and/or regionally adopted. National guidelines are mainly based on the international ones taking into consideration the existing recommendations and algorithms for treatment as well as the local practical experience. Comparing the national and international guidelines’ recommendations, we could identify the differences in the practices and analyze the reasons behind them, which in turn informs decision makers what the gaps are and how they could be remedied. On the other hand, guidelines are often used by reimbursement bodies to set criteria for initiating a patient on a specific therapy and thus, they could indirectly regulate the patient access to medicines. Some reimbursement institutions are posing additional limitations on advanced therapies prescribing with the aim of containing the probable financial impact.

Rare diseases (RDs) present a major financial concern and challenge for individual healthcare systems worldwide especially for low- and middle-income countries’ public funds and with restrictive budget policies, such as the Bulgarian one (Kamusheva et al., 2018a). Moreover, provision of the latest innovative and advanced therapies for patients with rare diseases is their human right as every other citizen’s (Human Rights Council, 2018). Development, implementation and update of pharmaco-therapeutic guidelines for RDs following the latest international therapeutic recommendations is crucial and necessary for practitioners, patients and decision makers. Clinical practice guidelines for RDs shorten the time to diagnosis, optimize the therapeutic decisions and lead to better outcomes (Wilson, 1997). Many European countries defined development of such guidelines as a main goal in their national plans on RDs (Rodwell and Aymé, 2014; Pavan et al., 2017).

Ensuring an adequate financial access to therapy through the reimbursement systems is another big challenge facing the health policies of each country. Sometimes, the access is worsened due to delay of market entrance of the products as a result of manufacturer’s marketing strategies, slow procedures or unstable legislative framework on a local level (Kamusheva et al., 2018a; Vassileva et al., 2019; Szegedi et al., 2018; NCPR, 2021). Health policy decision makers should overcome a number of barriers in order to provide high cost medicines despite the limited budgets (Wahlster et al., 2015). Therefore, the health policy should develop a country-specific set of measures for improving RDs patients’ access to innovative medicines. Such a measure would be the implementation of specific legislative requirements for clinical and economic assessment of these therapies, which take into account the international therapeutic guidelines and the best clinical practices. However, the variation of access to orphan, ultra-orphan medicines, and medicines for rare diseases could not be eliminated among the countries due to differences in reimbursement requirements and considerations (Kanters et al., 2018).

The main goal of the study is to assess guideline compliance when treating patients with RDs in Bulgaria in terms of date of market approval, inclusion in the PDL and subsequent access to orphan MPs. Objects of the study were the most cost-consuming rare diseases. Both national and guidelines issued by the relevant European professional associations concerning treatment strategies were evaluated and compared for those diseases.

## Materials and Methods

### Rare Diseases Costs Paid by the National Health Insurance Fund

The most cost-consuming rare diseases in Bulgaria were defined on the basis of the official National Health Insurance Fund (NHIF) reports published in 2017 (National Health Insurance Fund Official Reports for Number of Health-Insured Patients and Reimbursed Costs, 2019). Data regarding cost paid by the NHIF and the number of health-insured people with a particular rare disease was extracted and analyzed. The costs are presented in EUR (1 EUR = 1.95583 BGN).

### Evaluation of Patients’ Access to Medicines for Rare Diseases

The national pharmaco-therapeutic guidelines for treatment of the top 10 cost-consuming rare diseases were analyzed to assess their compliance with the guidelines issued by the relevant European professional associations. Guideline’s compliance index (GCI) was calculated for medicines for rare diseases available in the Positive Drug List (PDL) in Bulgaria using the following formula:

$$GCI=\frac{\text{Number of medicines available in the PDL in Bulgaria}}{\text{Number of medicines in the European guidelines}}$$

Compliance as a term is used to describe the level of similarity of the pharmaco-therapeutic guidelines adopted in Bulgaria and by the European professional associations regarding patterns for pharmaceutical treatment. The guidelines were also analyzed in respect to the recommended therapeutic outcomes and whether those outcomes have been used by the NHIF as criteria for patients’ inclusion on therapy.

Market entrance was evaluated through analysis of the availability of orphan medicines authorized through centralized procedure and available in the PDL and their date of inclusion since marketing authorization (MA). Early access scheme was not in the scope of the study as it requires more specific confidential information.

Patients’ access was defined as the number of reimbursed medicines included in the pharmaco-therapeutic guidelines and the time from marketing authorization to reimbursement decision by the national authorities.

Patient access was analyzed also through evaluation of the NHIF standards for OMs prescribing providing additional criteria for initiation and continuing treatment with the selected medicines. Descriptive statistics was used to calculate the average time, median and SD.

## Results

**FIGURE 1:**
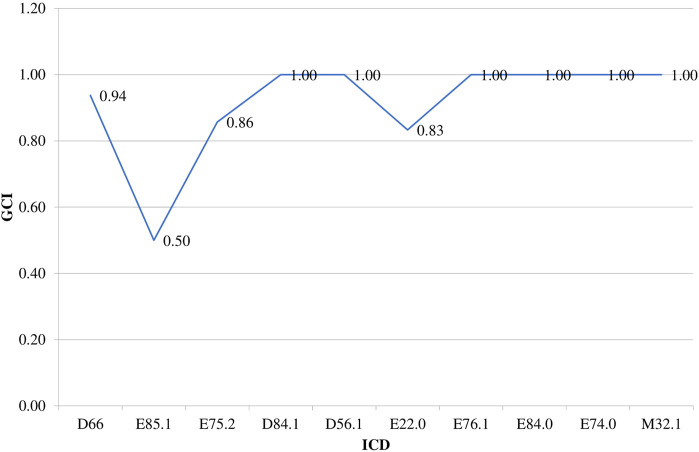
NHIF costs paid for RDs in 2017. Legend: Hereditary factor VIII deficiency (ICD D66), Neuropathic heredofamilial amyloidosis (E85.1), Other sphingolipidosis (Fabry, Gaucher, Niemann-Pick) (E75.2), Defects in the complement system (D84.1), Beta thalassemia (D56.1), Acromegaly and pituitary gigantism (E22.0), Mucopolysaccharidosis, type II (E76.1), Cystic fibrosis with pulmonary manifestations (E84.0), Glycogen storage disease (E74.0), other forms of systemic lupus erythematosus (M32.8).

All rare diseases (RDs) (121 in total) prevalent in the Bulgarian population are included in a specific list issued by the Minister of Health which is regularly updated. These diseases are covered with public funds by the NHIF (Order of Minister of Health, 2015; NCPR, 2021). The NHIF covered treatment for 70 RDs in 2017 with most of them being for Congenital malformations, deformations and chromosomal abnormalities (Q00-Q99) (20 out of 70) followed by Endocrine, nutritional and metabolic diseases (E00-E90) (17 out of 70). The first 10 most expensive RDs are Hereditary factor VIII deficiency (ICD D66), Neuropathic heredofamilial amyloidosis (E85.1), other - Sphingolipidosis (Fabry, Gaucher, and Niemann-Pick) (E75.2), Defects in the complement system (D84.1), Beta thalassemia (D56.1), Acromegaly and pituitary gigantism (E22.0), Mucopolysaccharidosis, type II (E76.1), Cystic fibrosis with pulmonary manifestations (E84.0), Glycogen storage disease (E74.0) and Other forms of systemic lupus erythematosus (M32.8) (ICD-10 Version, 2016) (Figure 1).

Table 1 presents the comparison between the Bulgarian and European guidelines regarding the respective pharmacotherapies for selected RDs, the year of their marketing authorization through centralized procedure in the European Union and the time of their inclusion in the reimbursement list in Bulgaria.

**TABLE 1:** Comparison of pharmaco-therapeutic guidelines and Bulgarian patients’ access to medicines for the top 10 most expensive RDs.

Active substance	Marketing authorization date	European guidelines	National guidelines	Availability of the medicine in Bulgaria (PDL)	Date of inclusion in PDL	Additional criteria of NHIF for patient access to therapy
D66 HEREDITARY FACTOR VIII DEFICIENCY						
*Human coagulation factor VIII*	*2015* *^a^*	*+*	*+*	*+*	*2015*	*+*
*Human coagulation factor VIII*	*1999* *^a^*	*+*	*+*	*+*	*2010*	*+*
*Human coagulation factor VIII*	—	*+*	*+*	*+*	*2010*	*+*
*Human coagulation factor VIII*	*2007* *^a^*	*+*	*+*	*+*	*2010*	*+*
*Recombinant Coagulation factor VIII (octocog alfa)*	*2004*	*+*	*+*	*+*	*2010/2015*	*+*
*Recombinant Coagulation factor VIII (octocog alfa)*	*2000*	*+*	*+*	*+*	*2009/2011*	*+*
*Recombinant Coagulation factor VIII (octocog alfa)*	*2016*	*+*	*+*	*+*	*2017*	*+*
*Recombinant Coagulation factor VIII (turoctocog Alfa)*	*2013*	*+*	*+*	*+*	*2015*	—
*Recombinant Coagulation factor VIII (simoctocog Alfa)*	*2014*	*+*	*+*	*+*	*2015*	—
*Recombinant Coagulation factor VIII (moroctocog alfa)*	*1999*	*+*	*+*	*+*	*2011/2015*	—
*Coagulation factor VIII (rurioctocog alfa pegol)*	*01.2018*	*+*	*+*	*+*	*12.2018*	—
*Coagulation factor VIII (efmoroctocog alfa)*	*2015*	*+*	*+*	*+*	*2017/2018*	*+*
*coagulation factor, Factor VIII inhibitor bypassing activity*	*2015* *^a^*	+	*+*	*+*	*2017*	*+*
*Coagulation factor VIII, Factor von Willebrand*	*2011* *^a^*	+	*+*	*+*	*2013*	*+*
*eptacog alfa (activated)*	*1996*	+	*+*	*+*	*2016*	*+*
*desmopressin acetate*	*^a^*	*+*	*+*	*−*	—	—
E85.1 NEUROPATHIC HEREDOFAMILIAL AMYLOIDOSIS						
*Tafamidis*	*2011*	*+*	*+*	*+*	*2015*	*+*
*Diflunisal*	—	*+*	*−*	*−*	—	—
*Patisiran*	*2018*	*−*	*−*	*−*	—	—
*Inotersen*	*2014*	*−*	*−*	*−*	—	—
E75.2 OTHER SPHINGOLIPIDOSIS						
*Imiglucerase*	*1997*	**+**	**+**	***+***	*2010*	*+*
*Eliglustat*	*2015*	*+*	*+*	*+*	*2018*	*+*
*Velaglucerase*	*2010*	*+*	*−*	*−*	—	—
*agalsidase alfa*	*2001*	*+*	*+*	*+*	*2014*	*+*
*agalsidase beta*	*2001*	*+*	*+*	*+*	*2011*	*+*
*Miglustat*	*2017*	+	*+*	*+*	*2019*	*+*
*Miglustat*	*2002*	+	+	*+*	*2011*	*+*
D84.1 DEFECTS IN THE COMPLEMENT SYSTEM						
*c1-inhibitor, plasma derived*	*2013* *^a^*	*+*	*+*	*+*	*2013*	*+*
*conestat alfa*	*2010*	*+*	*+*	*+*	*2012*	*+*
*Icatibant*	*2008*	*+*	*+*	*+*	*2018*	*+*
*immunoglobulin, normal human for extravascular adm*	*2010* *^a^*	*+*	*+*	*+*	*2012*	*+*
*immunoglobulins, normal human, for intravascular adm*	*2009* *^a^*	*+*	*+*	*+*	*2012*	*+*
D56.1 BETA THALASSAEMIA						
*Deferoxamine*	—	*+*	*+*	*+*	*2011*	*+*
*Deferiprone*	*1999*	*+*	*+*	*+*	*2011*	*+*
*Deferiprone*	*1999*	*+*	*+*	*+*	*2011*	*+*
*Deferasirox*	*2006*	*+*	*+*	*+*	*2018*	*+*
E22.0 ACROMEGALY AND PITUITARY GIGANTISM						
*Cabergoline*	—	+	+	−	—	—
*Bromocriptine*	*2002*	*+*	*+*	*+*	*2010*	—
*Bromocriptine*	*2001*	*+*	*+*	*+*	*2011*	—
*Pegvisomant*	*2002*	*+*	*+*	*+*	*2011*	*+*
*Octreotide*	*2000*	*+*	*+*	*+*	*2011*	*+*
*Pasireotide*	*2014*	*+*	*+*	*+*	*2017*	*+*
E76.1 MUCOPOLYSACCHARIDOSIS, TYPE II						
*Idursulfase*	*2007*	*+*	*+*	*+*	*2012*	*+*
E84.0 CYSTIC FIBROSIS WITH PULMONARY MANIFESTATIONS						
*Tobramycin*	*2016*	*+*	*+*	*+*	*2017*	*+*
*Tobramycin*	*2011*	*+*	*+*	*+*	*2012*	*+*
*colistimethate sodium*	*2012*	*+*	*+*	*+*	*2015*	*+*
*dornase alfa*	*1996* *^a^*	*+*	*+*	*+*	*2011*	*+*
*Ivacaftor*	*2012*	*+*	*+*	*−*	—	—
*lumacaftor/ivacaftor*	*2015*	*+*	*+*	*−*	—	—
E74.0 GLYCOGEN STORAGE DISEASE						
*alglucosidase alfa*	*2006*	*+*	*+*	*+*	*2013*	*+*
M32.1 SYSTEMIC LUPUS ERYTHEMATOSUS WITH ORGAN OR SYSTEM INVOLVEMENT; M32.8 OTHER FORMS OF SYSTEMIC LUPUS ERYTHEMATOSUS						
*Methylprednisolone*	*1993* *^a^*	*+*	*+*	*+*	*2013*	—
*Belimumab*	*2011*	*+*	*+*	*+*	*2013*	*+*
*Chloroquine*	*2001* *^a^*	*+*	*+*	*+*	*2016*	—

a Marketing authorization is not based on the centralized procedure.

The average time to access of orphan medicinal products (OMPs) in Bulgaria authorized through centralized procedure is 6.75 years (SD = 4.96). Median time is 6.5 years pointing out that the majority of orphan medicines have a time-lag between marketing authorization and reimbursement in Bulgaria. The longest time is observed for eptacog alfa (20 years); imiglucerase and agalsidase alfa (13 years); moroctocog alfa, deferiprone, deferasirox (12 years); octreotide (11 years); agalsidase beta and icatibant (10 years) and the shortest for rurioctocog alfa pegol, octocog alfa and simoctocog alfa (1 year), efmoroctocog alfa, conestat alfa, belimumab and miglustat (2 years). Bulgarian patients with cystic fibrosis with pulmonary manifestation had been waiting for only 1.6 years to get access to innovative centralized authorized medicines, whereas for patients with acromegaly, the time to adequate access to treatment was 8.2 years. Diflunisal is a nonsteroidal anti-inflammatory drug indicated for stage I and II of transthyretin familial amyloid polyneuropathy (TTR-FAP) according to the European consensus for diagnosis, management, and treatment of this disease (Adams et al., 2016). It is not included in the Bulgarian guidelines and is not marketed in the country. However, tafamidis which is currently indicated in the European Union for adult patients with TTR amyloidosis in stage I symptomatic polyneuropathy, and which could delay peripheral neurological impairment (Adams et al., 2016), is part of the national guideline for treatment of neurology disorders and paid by the NHIF (Pharmacotherapeutic guidea). Velaglucerase is approved in the EU and it is a part of the Gaucher disease therapy, but it is not available to Bulgarian patients (Biegstraaten et al., 2018). The other approved enzyme replacement therapy for patients with Gaucher disease, reimbursed in Bulgaria, is imiglucerase (Pharmacotherapeutic guideline for allergic diseases, 2019).

The national pharmaco-therapeutic guideline (Pharmacotherapeutic guideline for endocrinological diseases, 2019) is in accordance with the recommendations of the European guideline for treatment of rare factor deficiencies (Peyvandi and Menegatti, 2016) and the World Federation of Hemophilia guideline for management of hemophilia (Srivastava et al., 2013). The main treatment approach is to replace the deficient coagulation factor and to use adjunctive therapies if needed (Pharmacotherapeutic guideline for hematological diseases, 2019).

Hereditary angioedema (HAE) is a rare disorder also known as complement component 1 inhibitor deficiency. Recent international guidelines for its management include C1-esterase inhibitor (C1-INH) as an acute treatment option. C1-INH are recommended as first-line treatment for long-term prophylaxis and in case of short-term prophylaxis (Henry Li et al., 2019). Bulgarian patients with HAE are also provided with access to recombinant C1-INH formulations and plasma-derived products which are effective and well tolerated options (Pharmacotherapeutic guideline for neurological diseases, 2019).

Chelation therapy for patients with beta thalassemia is recommended by Thalassemia International Federation, United States, Canadian, United Kingdom, Italian and Australian guidelines as well as by the Bulgarian guideline for hematological diseases (Musallam et al., 2013; Pharmacotherapeutic guideline for rheumatological diseases, 2019). The initiating iron chelation therapy after particular number of transfusions or when a serum ferritin level >1,000 ng/ml. Deferasirox, deferiprone and deferoxamine are reimbursed and are available under different pharmaceutical formulations (tablets, oral solution, powder for solution for injection).

The European Society of Endocrinology Clinical Practice Guidelines for the management of aggressive pituitary tumors and carcinomas and the Bulgarian guideline for endocrinological diseases recommend first (octreotide) and second generations (pasireotide) somatostatin analogues as well as pegvisomant and dopamine agonist therapy alone or in addition to somatostatin analogue or pegvisomant (Raverot et al., 2018; Pharmacotherapeutic pneumology and physiatry guideline, 2019).

Enzyme replacement therapy (idursulfase) for patients with Hunter’s disease has been included as a main option which significantly improves somatic signs and symptoms (Whiteman and Kimura, 2017). Idursulfase has been available for Bulgarian patients and reimbursed since 2012.

Ivacaftor and combination of lumacaftor/ivacaftor are recommended for a group of cystic fibrosis patients with specific mutation both from the latest 2018 revision of European Cystic Fibrosis Society (ECFS) practice guideline and from the Bulgarian pulmonology guideline (Castellani et al., 2018; Pharmacotherapeutic pneumology and physiatry guideline, 2019). However, they still have not obtained a reimbursement status in Bulgaria.

A consensus statement regarding initiation and termination of ERT with alglucosidase alfa, available for patients with Pompe disease, was published in 2017. It is also part of the therapeutic strategy described in the Bulgarian pharmacotherapeutic neurological diseases guideline approved in 2018 (ICD-10 Version, 2016).

Belimumab is a biologic agent considered to be appropriate in case of persistently active systemic lupus erythematosus. It is included in the updated recommendations regarding the management of systemic lupus erythematosus (SLE) taking into account both scientific evidence and expert-opinion (Fanouriakis et al., 2019). Belimumab is part of the therapeutic schemes described in the Bulgarian rheumatology pharmacotherapeutic guideline (Pharmacotherapeutic guideline for rheumatological diseases, 2019).

**FIGURE 2:**
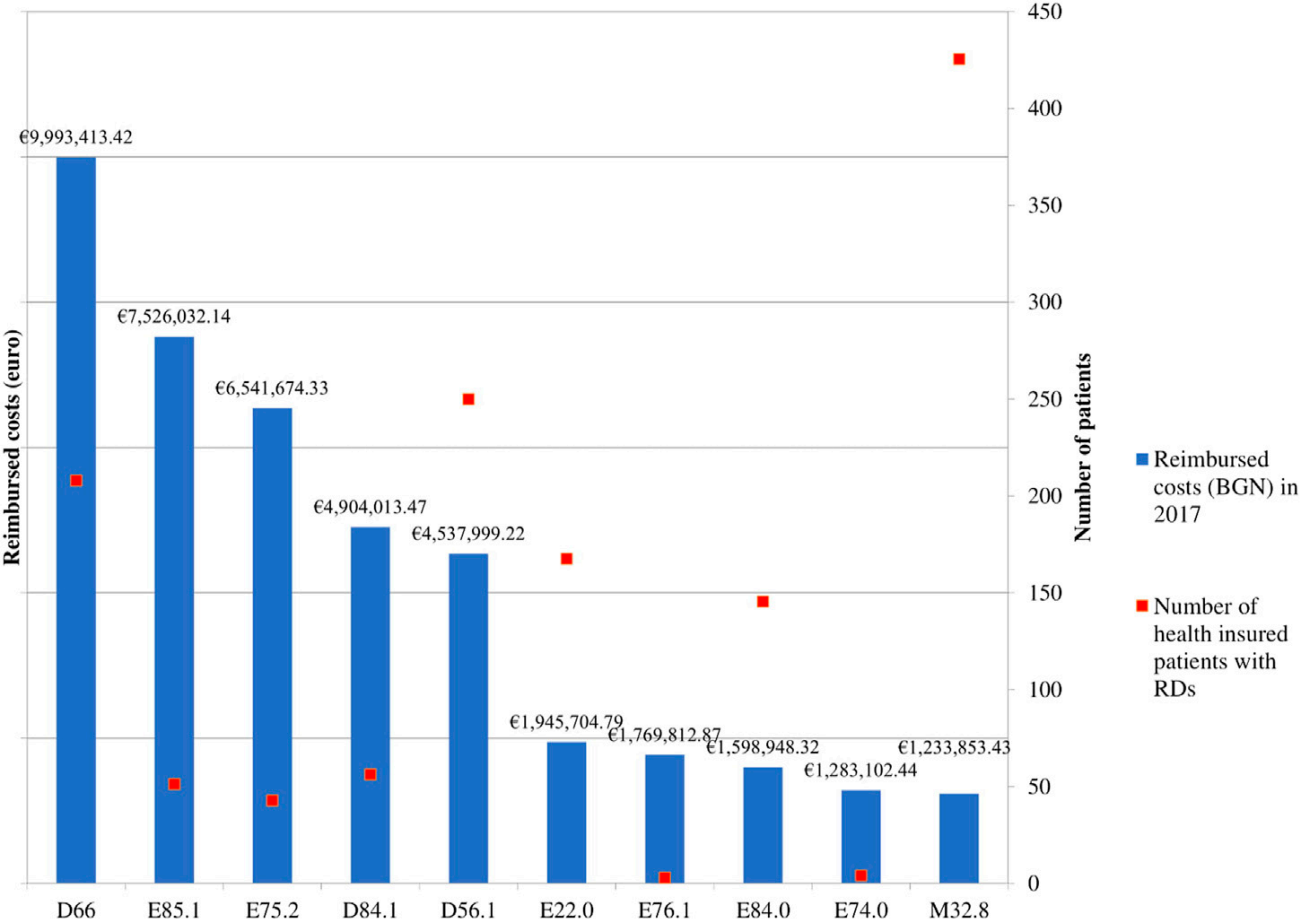
Guidelines compliance index (GCI). Legend: CD – International Classification of Diseases, 10th revision; GCI – Guidance Compliance Index, Hereditary factor VIII deficiency (ICD D66), Neuropathic heredofamilial amyloidosis (E85.1), Other sphingolipidosis (Fabry, Gaucher, Niemann-Pick) (E75.2), Defects in the complement system (D84.1), Beta thalassemia (D56.1), Acromegaly and pituitary gigantism (E22.0), Mucopolysaccharidosis, type II (E76.1), Cystic fibrosis with pulmonary manifestations (E84.0), Glycogen storage disease (E74.0), other forms of systemic lupus erythematosus (M32.8).

Guideline compliance index is presented in Figure 2. It is obvious that for a great number of evaluated rare diseases almost full similarity between the number and type of medicines included in the Bulgarian and international guidelines exists. The lowest guideline compliance index, equal to 0.5, is identified for Neuropathic heredofamilial amyloidosis (E85.1) as of 2017 only 1 medicine (tafamidis) out of 2 available in the guidelines was reimbursed in the country.

Specific requirements regarding initiating and continuing of therapy with all orphan medicines or medicines for the rare diseases selected in the current study and reimbursed by the NHIF are available and applied. They are officially published and could be considered as inclusion and exclusion criteria for treatment. Patients should be strictly followed-up on a particular period of time – usually every 6 months (National Health Insurance Fund Requirements for Treatment of Rare Diseases, 2019). These criteria specify the group of patients for whom the particular therapy is most appropriate and should be reimbursed (Table 1).

The monitored therapeutic outcomes described in the national guidelines comply with those included in the European guidelines and consensuses. They are related to disease pathophysiology and patients are strictly followed-up so as to assess the level of clinical improvement (Table 2). As evident from Table 2 **,** the national health insurance fund necessitates similar clinical evidences for therapy initiation as the European guidelines.

**TABLE 2:** Assessment of the main therapeutic outcomes for observed rare diseases.

	Bulgarian guidelines	European guidelines
INN	Clinical indicators and tests for following up and assessing the therapeutic outcomes	
Recombinant coagulation factors	Bethesda assay test, complete blood count (CBC), AST, ALT, Anti-HCV antibodies, HBsAg, HIV, medical imaging	Physical scores, imaging techniques, X-ray, MRI, quality of life, number of bleeds, severity of bleeds, joint ABR etc.
Tafamidis	Body mass, BMI, total protein, albumin, CBC, erythrocyte sedimentation rate (ESR), serum electrolytes, blood glucose, creatinine, urinalysis, arterial blood pressure, ECG	Health-related quality of life, cardiac biomarker N-terminal pro-hormone brain natriuretic peptide, echocardiographic parameters
Imiglucerase, eliglustat	Hematological, visceral (liver volume), skeletal, pulmonary criteria for achieving therapeutic effect + improving the quality of life	Hemoglobin levels, platelet counts, spleen and liver volumes, z scores for height and bone mineral density, and reports of bone pain and bone crises
Agalsidase alfa and beta	Blood pressure, CBC, total protein, AST, ALT, GGT, alkaline phosphatase, total cholesterol, TG, blood sugar, proteinuria, kidney biopsy, MRI, ECG	Kidney function, proteinuria, globotriaosylceramide levels, heart functions, QoL
Miglustat	Clinical parameters of neurological disease and neuropsychiatric assessment, hearing assessment, abdominal ultrasound, CBC, ASAT, ALT, CT or MRI, etc.	Neurological assessment, CBC
Deferoxamine, deferiprone, deferasirox	Serum ferritin values, cardiac and hepatic MRT, and left ventricular ejection fraction over a period of 6–12 months	Improvement in right ventricular ejection fraction; hepatic outcomes
Somatostatin analogues	IGF-1 levels and GH-levels	IGF-1 levels and GH-levels
Pegvisomant	IGF-1 levels	IGF-1 levels
Idursulfase	Body weight, height, head circumference, CBC, AST, ALT, study of glycosaminoglycan levels; abdominal ultrasound (liver and spleen sizes), EEG, 6-min walking test, ECG, echocardiography	Anti-idursulfase antibodies, vital signs, physical examination, 12-lead electrocardiogram, concomitant medications or procedures, laboratory testing (clinical chemistry, hematology and urinalysis), 6-min walking test
Dornase alpha; colistimethate sodium	Body weight, growth, CBC, ESR, FEV1; blood glucose, AST, ALT, microbiology, creatinine and urea in every 6 full-cycle therapy and at discretion, consultation with a neurologist, nephrologist, endocrinologist	Clinical assessment, microbiological assessment, microbiological assessment, lung function testing
Alglucosidase alfa	Every 6 months: Neurological status, manual muscular testing, functional breath test, assessment of daily life activities	Percent predicted forced vital capacity (% FVC), 6-min walk test (6 MWT)
Belimumab	Every 6 months: CBC with differential blood count (DKK), AST, ALT, creatinine, proteinuria, ANA and/or anti-dsDNA or other extractable antibodies	SLE severity and anti-dsDNA antibody titers, renal outcomes

Anti-dsDNA - anti-double stranded DNA; ALT - alanine aminotransferase; AST - aspartate aminotransferase; BMI – body mass index; CBC – complete blood count; ECG - Electrocardiography; ESR - sedimentation rate of erythrocytes; FEV1 - forced expiratory volume in 1 s; FVC - Forced vital capacity; GGT - gamma-glutamyl transferase; GH – growth hormone; HBsAg - Hepatitis B Virus Surface Antigen; HIV – human immunodeficiency virus; IGF-1 - Insulin-like growth factor 1; MRI - Magnetic resonance imaging; MRT - Magnetic Resonance Tomography; SLE - Systemic lupus erythematosus.

## Discussion

The current study revealed a significant variation in the time between market entrance and respective access to different medicines treating a particular rare disease. То some extent, it also confirms the results from a previous study, which revealed that Bulgarian patients have a relatively delayed access to innovative medicines, some of them for rare diseases (Kamusheva et al., 2018b), in comparison to other countries. The average time from MA to a reimbursement decision for orphan medicines in Italy, France and Spain is 18.6, 19.5 , and 23.0 months, respectively (Zamora et al., 2019). In Germany, reimbursement occurs immediately after marketing authorization (MA), while in England less than 50% of centrally-approved orphan medicines are funded by the National Health Service (Zamora et al., 2017). Zamora et al. discussed these differences with the early access schemes which ensure shorter time to access to orphan medicines. Such early access schemes had not been available in Bulgaria until 2019 and the implementation of a specific text in the national pharmaceutical legislation regarding the so called “compassionate use.” The effect should be examined in further studies.

Guidelines’ compliance index showed a significant overlap between the type and number of medicines included in the therapeutic schemes in Bulgaria and in Europe. All medicines for defects in the complement system, beta thalassemia, mucopolysaccharidosis type II, cystic fibrosis with pulmonary manifestations, glycogen storage disease and forms of systemic lupus erythematosus described in the European guidelines are available in the Bulgarian one and are reimbursed by the national public fund. Following and adopting the European pharmaco-therapeutic guidelines is a result of the attempts to ensure the most appropriate and innovative pharmacotherapy for the Bulgarian patients. Bulgarian guidelines are a product of joint activities between medical specialists, patient organizations and regulatory bodies. Moreover, such joint efforts could be explained with the limited number of therapeutic options which are crucial to be ensured for all indicated RDs patients.

Our study adds more information about the way of application of European guidelines for RDs therapy on a national level, more specifically, the Bulgarian guidelines and reimbursement practice. This is the first national study comparing the national and international pharmaco-therapeutic guidelines on rare diseases and their influence on the patients’ access to therapy. It shows that, at the point of the analysis, the reimbursement policy in Bulgaria is restrictive and very cautious in using clinical guidelines when admitting patients’ access to therapy. For some of the medicines the access to market is extremely delayed but majority manage to get access within 6.5 years as a result of the restrictive policy Some other factors that might affect the time to access could be the specific national legal requirements for pricing and reimbursement decisions, population of interest and manufacturers intentions to enter certain markets. (Tsekov et al., 2021).

A strong limitation of the current analysis is that the date to market entrance could not be accurately found for medicines authorized before 2007 when pharmaceutical legislation changes were implemented due to Bulgarian membership in the European Union. Another limitation and probably an object of further study is performing a more detailed analysis of the therapeutic indicators and procedures for initiation and continuing of treatment, as well as on the additional limitations for OMs prescribing to patients. What we have revealed in the current pilot analysis is a partial similarity of these indicators between Bulgaria and the adopted European recommendations and availability of strict criteria for patients’ access to innovative therapy in Bulgaria. Bulgarian clinical practice, with the assistance of health policy decision makers and expert, attempts to follow the European and international clinical and pharmacotherapeutic guidelines and to provide Bulgarian patients with RDs to innovative therapies.

To the best of our knowledge, this is one of the few studies which attempt to analyze the Bulgarian RDs patients’ access to therapy applying a set of instruments: direct comparison between the pharmacotherapeutic guidelines on national and international level; assessing the time of inclusion and receiving reimbursement status after marketing authorization in the EU; analyzing the availability of national standards for initiation and continuing of treatment. One other study has evaluated the time between market and patients’ access to breast cancer therapy and compliance with international guidelines (Dimitrova et al., 2020). The results show that most of the therapies are covered with public finances and the average time from marketing authorization to market and patients’ access is 1–2 years on average. It also showed that there is a need for stricter compliance and regular updates of national to the international guidelines (Dimitrova et al., 2020). Further studies are planned aimed at more detailed and deeper analysis and comparison covering other rare diseases.

## Conclusion

Treatment of rare diseases in Bulgaria mostly follows the European guidelines. The main factors influencing the market entrance and patient access are the time to inclusion in the PDL and related requirements and the NHIF criteria for selection and follow-up the patients which could be considered as restrictive ones focused on those patients who most need the therapy.

## Data Availability

The raw data supporting the conclusion of this article will be made available by the authors upon reasonable request.
